# A Facile Alkali-Assisted Synthesis Strategy for Hierarchical Porous Carbon Aerogels for Supercapacitors

**DOI:** 10.3390/molecules29225413

**Published:** 2024-11-16

**Authors:** Huimin Yang, Mingfang Zhang, Xinwei Guan, Xiaogang Shang, Lingfeng Zhu, Haimei Xu, Songbo Li

**Affiliations:** 1 School of Chemistry and Chemical Engineering, Inner Mongolia University of Science & Technology, Baotou 014010, Chinashangxiaogang2022@163.com (X.S.); 2 Centre for Atomaterials and Nanomanufacturing (CAN), School of Science, RMIT University, Melbourne, VIC 3000, Australia; xinwei.guan@rmit.edu.au (X.G.); lingfeng.zhu@rmit.edu.au (L.Z.)

**Keywords:** carbon aerogels, hierarchical porous carbon, energy storage, capacitor

## Abstract

Carbon aerogels synthesized via the polymerization of resorcinol (R) and formaldehyde (F) exhibit remarkable physiochemical properties, such as high thermal stability and excellent electrical conductivity. However, their limited specific surface area and porosity restrict their application potential. Herein, we developed hierarchical porous carbon aerogels using a one-step carbonization and activation method, directly converting the resin into carbon aerogel material by adding KOH as an activating agent. In contrast to conventional carbon aerogels with an irregular block ground structure, our hierarchical porous carbon aerogels exhibit substantially enhanced specific surface area, total pore volume, and surface oxygen content. In addition, this straightforward one-step fabrication approach holds significant promise for energy storage applications. Notably, the hierarchical porous carbon aerogel C1, with a KOH/RF mass ratio of 1, was proven to be the most effective electrode candidates, achieving a specific capacitance of 261.9 F·g^−1^ at 1 A·g^−1^ and 208.2 F·g^−1^ at 20 A·g^−1^. Moreover, it exhibited an outstanding rate capability of 79.5% and excellent capacity retention of approximately 97.5% after 10,000 cycles (7 A·g^−1^). This work highlights a promising approach for synthesizing commercial-grade carbon aerogels with hierarchical porosity, enabling high-performance energy storage applications.

## 1. Introduction

Highly efficient energy storage devices have received extensive attention due to the high demand for sustainable energy in modern society [1,2]. Supercapacitors have exhibited immense potential in energy storage and conversion technologies because of their high power density and excellent cyclic performance [3,4,5]. It is well known that the performance of electrochemical capacitors depends on the physiochemical properties of electrode materials, such as porosity, conductivity, and stability [6]. In recent years, carbonaceous materials such as activated carbon [7], graphene [8], aerogel carbon [9], and carbon fibers [10] have been widely explored as supercapacitor electrode materials due to their good electrical conductivity, high electrochemical stability, and large specific surface area. Among these, hierarchically porous carbon aerogel materials stand out as promising candidates thanks to their porous structures, effectively enhancing the efficiency of ion penetration [11,12,13]. Consequently, the development of carbon aerogels with superior porosity structures has become a critical focus in the supercapacitor.

To date, carbon aerogels are typically referred to resorcinol formaldehyde (RF), which are synthesized by polymerization of resorcinol and formaldehyde [14]. The resulting three-dimensional, cross-linked hierarchical porous structures of carbon aerogels derived from their inherent nature are highly favored for their excellent capacitive performance and rate capability [15]. In order to maintain the integrity of these pore structures, a large amount of research has been devoted to the improvement of synthesis methods. Factors such as types of templates, drying conditions, and carbonization or activation processes significantly influence the porosity, morphology, and surface area of the resulting materials [16,17,18].

The first protocol requires the use of hard templates with well-defined structures, such as silicon dioxide and metal oxides [19]. Nevertheless, the synthesis and subsequent removal of templates are both difficult and time-consuming. Alternative methods, including solvent exchange, supercritical CO_2_ drying, or freeze drying, are complex, costly, and energy-intensive because of long preparation procedures and the requirement for expensive and specialized equipment [14,20,21]. These challenges hinder the scalability of carbon aerogel production for industrial applications. Moreover, in most cases, carbonization and activation are usually carried out in two or more steps, and this is the main reason for low carbonization yield, and multi-step operation and the use of templates for carbon aerogels increase environmental pollution and time consumption. Therefore, further improvements in the synthesis of carbon aerogel are urgently needed to make the process more efficient and scalable.

With these considerations in mind, herein, we developed a one-step alkali-assisted carbonation and activation process to synthesize hierarchical carbon aerogels. In this strategy, a polymer gel serves as the carbon source, and KOH acts as the activating reagent. This facile approach simplifies the synthesis process and eliminates the need for templates, solution exchange, and supercritical drying processes, minimizing environmental pollution and cost consumption. The physicochemical properties of the prepared hierarchical carbon aerogel were evaluated thoroughly via Powder X-Ray Diffraction (XRD), Scanning Electron Microscopy (SEM), nitrogen adsorption–desorption measurement, and Raman Spectra and X-Ray Photoelectron Spectroscopy (XPS) tests. Next, its electrochemical performance of capacitance and capability and its long-cycling stability were further evaluated, making it a potential electrode material in supercapacitor applications.

## 2. Results and Discussion

**Figure 1:**
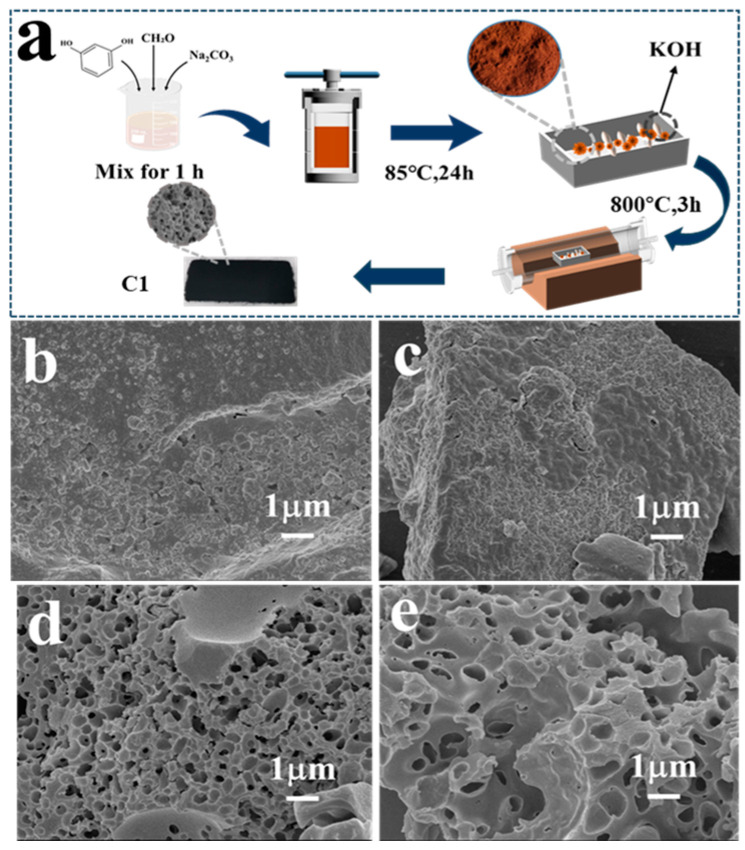
The synthesis process of carbon aerogel materials (**a**) and the SEM images of synthesized carbon aerogels materials with various KOH/RF ratios: (**b**) C0, (**c**) C0.5, (**d**) C1, and (**e**) C2.

The synthetic pathway for the formation of carbon aerogel is illustrated in Figure 1a. A mixture of certain amounts of resorcinol (C_6_H_6_O_2_), formaldehyde (CH_2_O), and NaCO_3_ was transferred to a stainless-steel hydrothermal reactor and further heated at 85 °C for 24 h. Then, the obtained power, mixed with a certain amount of KOH, was calcined in an inert atmosphere.

The obtained powder was repeatedly washed with deionized water before use. The SEM image of carbon material without the addition of KOH (Figure 1b) reveals aggregates of primary particles with only minimal pore structures on its surface. After adding a small amount of KOH during the activation process, the SEM image shows a surface structure similar to that without KOH, indicating that a small amount of KOH does not cause significant changes in the structure (Figure 1c). In contrast, the other samples prepared with high KOH/RF ratios (1 and 2) exhibit pronounced developed porous structures (Figure 1d,e). This is due to the fact that KOH, under high-temperature conditions, corrodes the surface of the carbon material, transforming its originally dense structure into a hierarchically porous state. A moderate KOH/RF ratio of 1 results in the formation of uniform and abundant pores on the material. These interconnected pores are expected to enhance the capacitive performance of the material [6].

**Figure 2:**
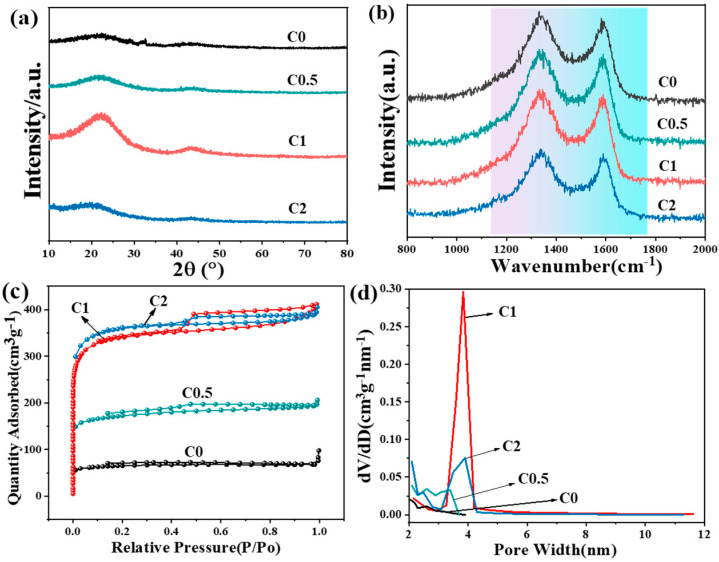
(**a**) XRD pattern and (**b**) Raman spectra. (**c**,**d**) Nitrogen adsorption–desorption isotherm and the pore size distribution of four samples, respectively.

The XRD patterns of the synthesized carbon aerogel materials (Figure 2a) are characteristic of amorphous carbons with graphitic domains (sp^2^ domains). The two broad diffraction peaks at approximated 22° and 43° were indexed to the (002) and (100) planes of graphite hexagonal structure, respectively [22]. This phenomenon is in agreement with that reported in the literature for carbons obtained from biomass [22]. Raman spectra were employed to detect the carbonization behavior and symmetrical vibration of carbon aerogels. As shown in Figure 2b, the D-band (disorder or defect carbonaceous structure) and G-band (ordered graphite in-plane vibrations) are located at 1339 cm^−1^ and 1592 cm^−1^, respectively. The intensity ratio of these two bands (I_D_/I_G_) is a diagnostical indicator for the degree of disorder and defect in carbon aerogels, and the results are given in Supplementary Table S1. With the increase in the KOH/RF ratio from 0.5 to 2, the ratios of I_D_/I_G_ elevated from 1.03 to 1.09, implying the presence of abundant structural defects. The result indicated that alkali etching ascribes to the structural changes in carbon materials. More alkali further facilitates the generation of amorphous carbon.

The specific surface area and pore size distribution of all samples were analyzed using nitrogen adsorption–desorption isotherms, as shown in Figure 2c,d and summarized in Table 1. All samples, except for C0, exhibited high uptakes at P/P_0_ < 0.05, indicating the presence of substantial micropores. The hysteresis loops observed in samples C0.5, C1, and C2, characterized by a typical H3 type over a wide pressure range of P/P_0_ = 0.4–1.0, reveal the formation of mesopores resulting from alkali activation. Notably, the mesopore size distributions for samples C1 and C2 are centered at ~4 nm, as illustrated in Figure 2d.

Additionally, a sharp increase in adsorption at relative pressures close to P/P_0_ = 1.0 for samples C1 and C2 suggests the presence of macropores, consistent with observations from SEM analysis. Together, these findings confirm the hierarchical pore structure in samples C1 and C2, comprising micropores, mesopores, and macropores. In particular, sample C1, activated with an optimal KOH concentration, achieved the highest mesopore content. This result is likely due to the etching effect of the increased KOH amount, where KOH facilitates structural modification by allowing K^+^ ions to penetrate the carbon pore walls, thus creating a unique porous network [23]. However, an excessive KOH concentration can disrupt the micro-mesopore structure, often leading to the formation of an amorphous structure.

Table 1 presents the BET surface area and pore parameters for all samples. The BET surface area of samples increased significantly with the elevated KOH/RF ratio during activation, with the 1151 and 1220 m^2^·g^−1^ of specific surface area obtained on C1 and C2. In addition, compared to C2 with excess KOH, C1 showed the highest mesoporous volume (0.167 cm^3^·g^−1^) and total pore volume (0.637 cm^3^·g^−1^). In contrast, the bulk carbon material without the addition of KOH during RF activation exhibited the lowest S_BET_ of 220 m^2^·g^−1^.

**Figure 3:**
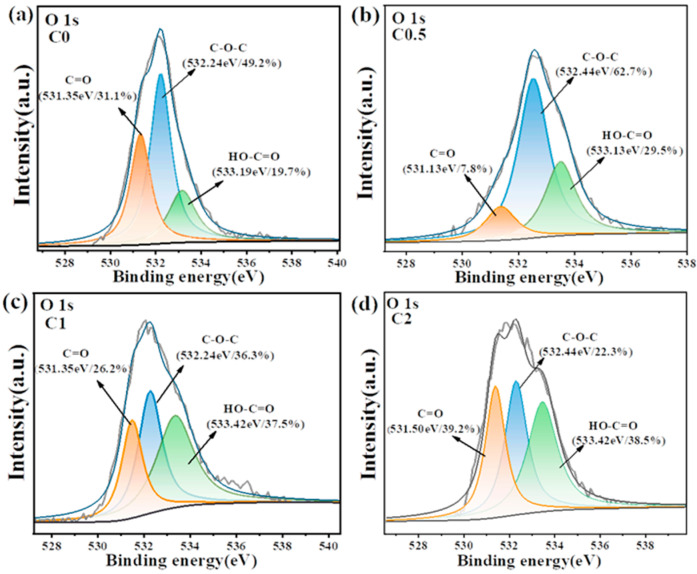
XPS spectra of O1s for C0 (**a**), C0.5 (**b**), C1 (**c**), and C2 (**d**), respectively.

Supplementary Figure S1 shows the XPS spectra of C0, C0.5, C1, and C2, indicating that all samples contain elements C and O, with corresponding peaks primarily represented by O 1s and C 1s peaks, respectively. The O 1S XPS spectra (Figure 3) of these four samples showed three obvious peaks at 531.13, 532.45, and 533.42 eV, which are attributed to C=O, C-O-C, and HO-C=O bonds, respectively [24,25].

The contents of HO-C=O bonds on carbon aerogels increased after KOH activation, which could be due to the alkali-promoted pyrolysis of C-O-C and/or the bond formation between carbon aerogels and KOH during activation. Supplementary Figure S2 displays the high-resolution C1 spectrum of the sample, with binding energies at 284.7, 285.9, 287.1, and 289.9 eV, corresponding to C-C, C-O, C=O, and HO-C=O bonds [26]. The increase in oxygen-containing functional groups was further evidenced by the FTIR spectrum (Supplementary Figure S3), where broad bands around 1187 cm^−1^ were observed, attributing to the stretching vibrations of C-O bonds in phenols, alcohols, or ethers [27,28].

**Figure 4:**
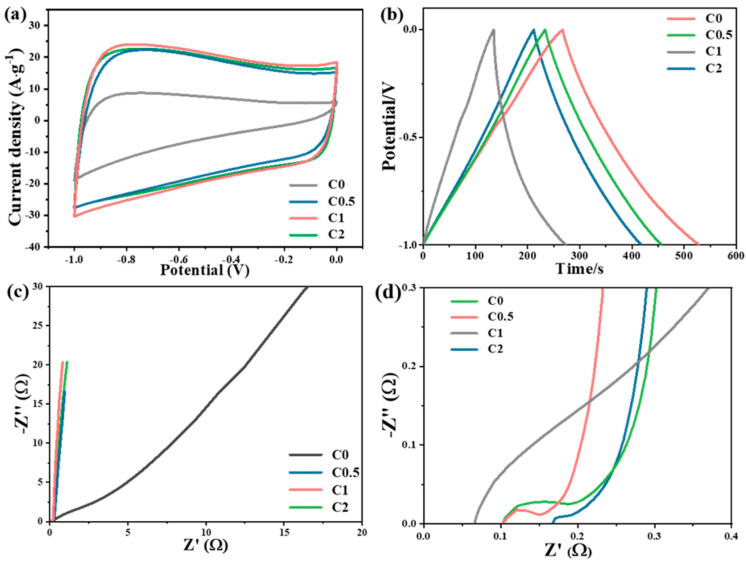
(**a**) CV curves of all samples at different scan rates, (**b**) GCD curves, (**c**) Nyquist plots, and (**d**) the magnified high-frequency region of the Nyquist plots.

With their high surface areas and hierarchical porous structures, the prepared carbon aerogel materials were subjected to study their potential capacitance properties as electrodes. The CV curves of all materials (Figure 4a) tested at a scan rate of 100 mV/s exhibited a rectangular shape, characteristic of the ideal electrical double-layer capacitive behavior. The linear and triangle-type GCD curves measured at a current density of 1 A·g^−1^ (Figure 4b) further confirmed the good capacitive performance [29]. The calculated specific capacitance from the GCD curves was 138, 205.8, 261.9, and 223.1 F·g^−1^ for carbon aerogels synthesized with a KOH/RF ratio of 0, 0.5, 1, and 2, respectively. It was noted that with the increase in the amount of KOH addition during activation, the capacitance exhibited a volcanic trend. The C1, with the mediate KOH amount, showed the maximum capacitance of 261.9 F·g^−1^. Furthermore, the C1 aerogel also exhibited a high rate capability of 261.9 A·g^−1^ at 1 A·g^−1^ and 208.2 g^−1^ at 20 A·g^−1^ (Supplementary Figure S4). This could be due to its large S_BET_ and pore size, which are key factors for enhancing ion diffusion, enabling high power and energy densities in supercapacitor applications [30]. A higher surface area could provide larger sites for ion accumulation and enhance the charge-transfer capability by reducing the contact resistivity between the electrolyte and the electrode. In addition, the abundant oxygen functional groups, particularly HO-C=O on C1, could considerably improve the electrolyte infiltration and the overall electrochemical performance of the electrode [7,31].

Electrochemical impedance spectroscopy (EIS) was performed to further evaluate the electrochemical performance of the four materials (Figure 4c,d). The steep slope in the low-frequency region demonstrates their capacitive performance with low Warburg resistance (Z_w_), and the semicircle in the high-frequency region demonstrates their charge-transfer resistance (R_ct_) [32]. C0 exhibited higher Warburg resistance (Z_w_), and C0.5 displayed higher internal resistance (R_Ω_). Conversely, C1 and C2 possessed more favorable resistance values than other samples, implying that their porous structure was beneficial for the charge-transition efficiency.

**Figure 5:**
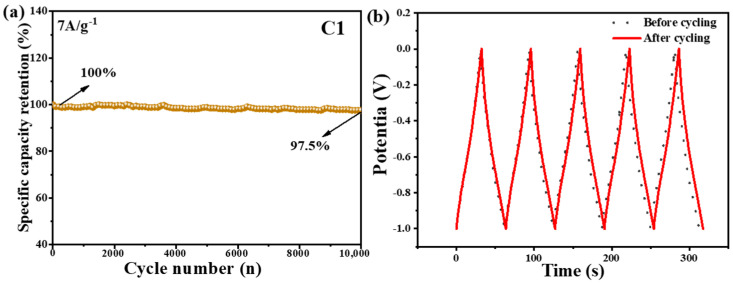
(**a**) The long cycling performance test of C1 at 7A·g^−1^ (**b**) and a comparison GCD test diagram of C1 sample before and after 10,000 cycles.

**Figure 6:**
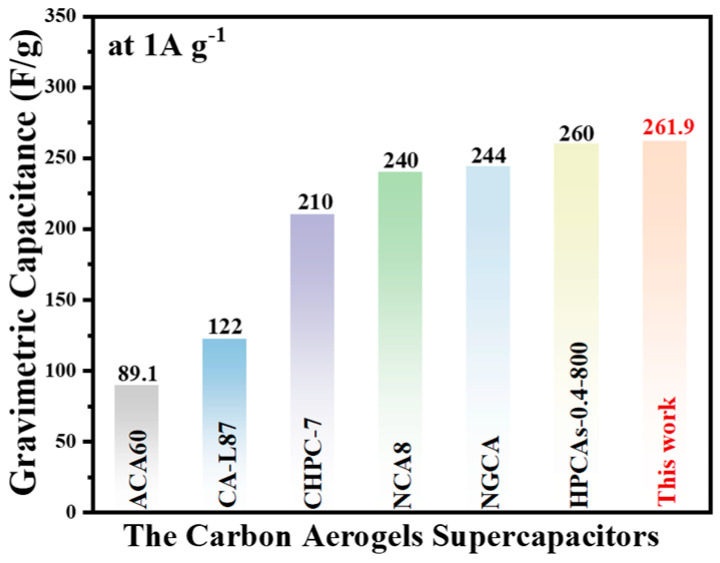
Comparison of the electrochemical performance between the C1 electrode and other previously reported related materials.

The cycling stability of electrode materials is important for their practical application in energy storage devices. The cycling performance of electrodes consisting of C1 was investigated by charging and discharging the capacitor 10,000 times at a current density of 7 A·g^−1^, as shown in Figure 5a. After 10,000 cycles, the specific capacitance only loses 2.5%. In Figure 5b, after 10,000 cycles, the specific capacitance only showed a 2.5% decrease, suggesting the good cycling stability of C1. These results indicate that the C1 electrode exhibits excellent cycle stability and capacity retention, making it a potential candidate for high-performance battery electrode materials. In addition, the C1 electrode presents a comparatively good electrochemical performance comparable to that of related published materials, as shown in Figure 6 and Supplementary Table S2 [33,34,35,36,37,38].

## 3. Materials and Methods

### 3.1. Synthesis of Hierarchical Carbon Aerogels

Hierarchical porous carbon aerogels were prepared via polymerization of resorcinol and formaldehyde, followed by carbonization and activation with alkali in one step. Typically, resorcinol (R, 2.20 g) was first dissolved in water (9 mL), and then formaldehyde (F, 3.25 g) and Na_2_CO_3_ (C, 22.1 mg) were added. The mixture was vigorously stirred for 1 h to form a homogeneous solution. This solution was then transferred to a 150 mL hydrothermal reactor, which was sealed and maintained at 85 °C for 24 h. Afterward, the mixture was cooled to room temperature, and the as-obtained wet gels were dried at 80 °C overnight.

For carbonization, the dried polymer gel was mixed with KOH at a mass ratio of 1:1 and carbonized at 800 °C for 3 h, with a heating rate of 5 °C/min. The carbon material was ultrasonically washed with distilled water until neutral and then dried at 80 °C overnight to obtain the final carbon aerogel material, denoted as C1. For comparison, samples without any base and with KOH to RF mass ratios of 0.5:1 and 2:1 were also prepared, and they are denoted as C0, C0.5, and C2, respectively.

### 3.2. Characterizations

Scanning electron microscopy (SEM; JSM-6701F, JEOL, Tokyo, Japan) was used to examine sample morphologies. The powder X-ray diffraction (XRD) patterns of the samples were recorded with a Stoe STADI P automated transmission diffractometer instrument equipped with an incident beam curved germanium monochromator selecting Cu Kα1 radiation. The XRD diffraction patterns were scanned in the 2θ range of 10–80°. The Raman spectra were recorded with a LabRAMHR Evolution (HORIBA JobinYvon, Paris, France) using a wavelength laser of 532 nm. Nitrogen adsorption–desorption isotherms were recorded with an ASAP2460 adsorption analyzer at 77 K (Micromeritics, Atlanta, GA, USA). The pore-size distribution from the desorption isotherm was calculated using the Barrett, Joyner, and Halenda (BJH) method. X-ray photoelectron spectroscopy (XPS) tests were performed on a PHI ESCA-5000C electron Spectrometer (ULVAC-PHI, Chigasaki, Japan).

### 3.3. Electrochemical Performance

The electrochemical properties of the samples were tested at a CHI660E electrochemical workstation (Chenhua, Shanghai, China) using a three-electrode system. For the preparation of the working electrode, a mixture of carbon aerogel, polyvinylidene difluoride (PVDF), and carbon black with a mass ratio of 8:1:1 was first sonicated in ethanol (EtOH) for 0.5 h, and the slurry was then cast onto a 1 × 1 cm^2^ of nickel foam. After drying in an oven at 60 °C for 24 h, the electrode was compressed under a pressure of ~10 MPa. A Hg/HgO and a Pt foil were used as the reference and counter electrodes, respectively.

The electrochemical performance of the working electrode was evaluated using cyclic voltammogram (CV), galvanostatic charge–discharge (GCD), and electrochemical impedance spectroscopy (EIS). A KOH (6 M) solution was used as the electrolyte, and the amount of active substance on the nickel foam was ~5 mg. The gravimetric specific capacitance (C_g_) of the electrodes was calculated using the following Equation (1) based on the discharge curves.

(1) $$C_{g}=\frac{I\times D_{t}}{m\times D_{v}}$$

In this equation, I(A) is the galvanostatic discharge current, D_t_ (V) is the discharge time, m (g) is the mass of the active material, and D_v_ (V) is the operating potential window.

## 4. Conclusions

In conclusion, we developed a facile alkali-assisted activation approach for fabricating hierarchical porous carbon aerogels without requiring templates, solution exchange, and supercritical drying. In this approach, hierarchical carbon aerogels are easily achieved through a one-step carbonation and activation process by adding KOH to the wet carbon materials prepared from resorcinol (R) and formaldehyde (F). This strategy not only simplifies the synthesis process but also reduces the environmental impact, as well as time and cost consumption. This finding shows that the KOH/RF ratio is a crucial factor in controlling the properties of the hierarchical porous carbon aerogels. An optimal KOH/RF ratio significantly enhances the specific surface area and pore structure, facilitating improved electrolyte diffusion and electron transport to active sites. Consequently, the as-prepared hierarchical C1 (KOH/RF = 1) with abundant mesopores exhibited an outstanding electrochemical performance as a supercapacitor, achieving a remarkable specific capacitance of 261.9 F·g^−1^ at the current density of 1 A·g^−1^ in 6 M KOH. More importantly, it demonstrated an excellent rate capability and maintained ~97.5% of its capacity after 10,000 cycles at a current density of 7 A·g^−1^. This work presents a promising and straightforward strategy for fabricating hierarchical porous carbon aerogels with potential applications in advanced supercapacitors, dye adsorption, batteries, CO_2_ adsorption, and beyond.

## Figures and Tables

**Table 1:** The structural properties of the four samples.

Sample	S_BET_ (m^2^·g^−1^)	V_meso_ (cm^3^·g^−1^)	V_total_ (cm^3^·g^−1^)	D_BJH_ (nm)
C0	220	0.0114	0.105	2.6
C0.5	582	0.0789	0.299	3.5
C1	1151	0.167	0.637	3.8
C2	1220	0.128	0.600	3.9

## Data Availability

The raw data can be provided upon reasonable request.

## Supplementary Materials

The following supporting information can be downloaded at https://www.mdpi.com/article/10.3390/molecules29225413/s1, Figure S1: The XPS spectra of C0, C0.5, C1, and C2; Figure S2: (a–d): The high-resolution C 1s spectrum of C0, C0.5, C1 and C2; Figure S3: The infrared spectra of C0, C0.5, C1, and C2; Figure S4: Specific capacitance of C0 and C1 at various current densities; Table S1: The intensity ratio of ID to IG in Raman spectra; Table S2: Comparison of the electrochemical performance between the C1 electrode and other previously reported related carbon aerogel materials.
