# Supplementary material for: A Facile Alkali-Assisted Synthesis Strategy for Hierarchical Porous Carbon Aerogels for Supercapacitors
Source: Molecules. 2024 Nov 16;29(22):5413. doi: 10.3390/molecules29225413 (PMC11597094; doi:10.3390/molecules29225413)
Supplement: Supplementary file 1 [file molecules-29-05413-s001.zip › molecules-3331745-supplementary.pdf]

Supplementary Information

A Facile Alkali-Assisted Synthesis Strategy for Hierarchical Porous Carbon Aerogels for Supercapacitors

Huimin Yang [1,*], Mingfang Zhang [1], Xinwei Guan [2], Xiaogang Shang [1], Lingfeng Zhu [2], Haimei Xu [2,*] and Songbo Li [1,*]

[1] School of Chemistry and Chemical Engineering, Inner Mongolia University of Science & Technology, Baotou 014010, China; zhangmingfang2024@163.com (M.Z.); shangxiaogang2022@163.com (X.S.)
[2] Centre for Atomaterials and Nanomanufacturing (CAN), School of Science, RMIT University, Melbourne, VIC 3000, Australia; xinwei.guan@rmit.edu.au (X.G.); lingfeng.zhu@rmit.edu.au (L.Z.)
* Correspondence: emma920@imust.edu.cn (H.Y.); haimei.xu@rmit.edu.au (H.X.); lisongbo@imust.edu.cn (S.L.)

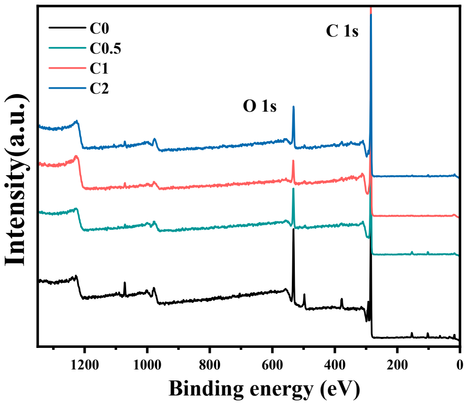

Figure S1. The XPS spectra of C0, C0.5, C1, and C2

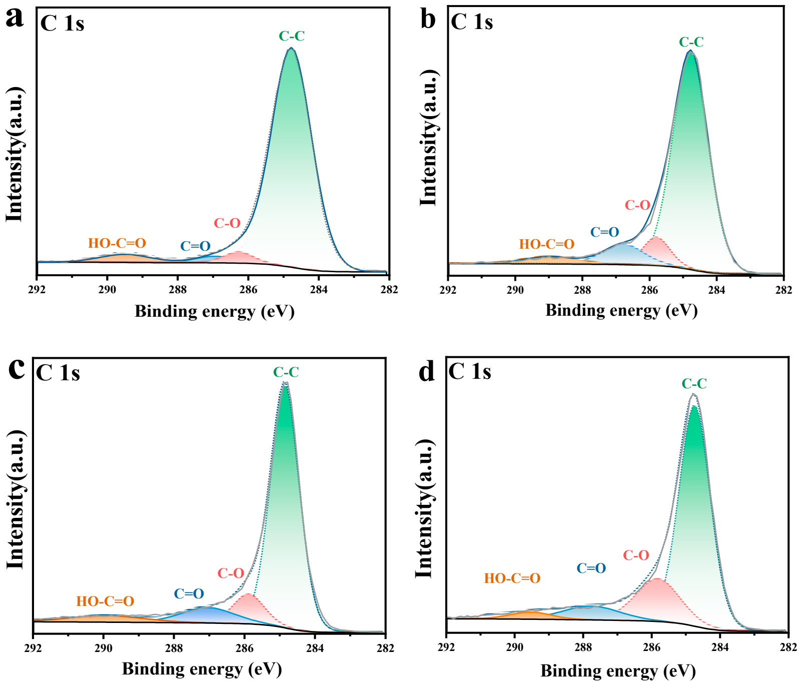

Figure S2. (a-d) The high-resolution C 1s spectrum of C0, C0.5, C1 and C2.

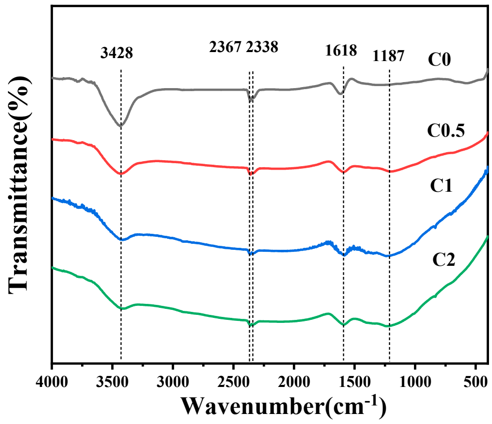

Figure S3. The infrared spectra of C0, C0.5, C1, and C2.

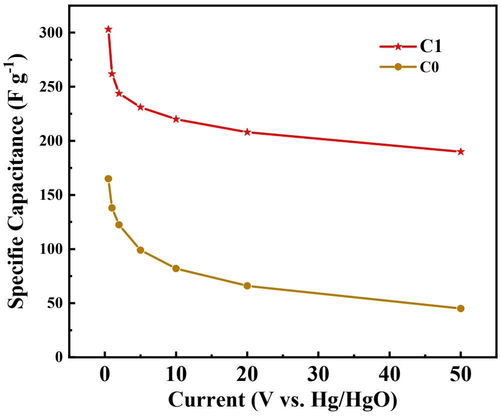

Figure S4. Specific capacitance of C0 and C1 at various current densities.

Table S1. The intensity ratio of I_D to I_G in Raman spectra.

Entry	Catalyst	I_D	I_G	I_D/I_G
1	C0	196	194	1.01
2	C0.5	200	195	1.03
3	C1	206	194	1.06
4	C2	144	132	1.09

Table S2. Comparison of the electrochemical performance between the C1 electrode and other previously reported related carbon aerogels materials.

Water electrolyzer	Specificcapacitance(Fg^{-1})	Currentdensity(Ag^{-1})	Electrolyte	Reference
ACA60	89.1	1	1M H2SO4	[1]
CA-L87	122	1	6M KOH	[2]
CHPC-7	210	1	6M KOH	[3]
NCA8	240	1	6M KOH	[4]
NGCA	244	1	6M KOH	[5]
HPCAs-0.4-800	260	1	6M KOH	[6]
C1	261.9	1	6M KOH	This work

1. Lee, J.-H.; Lee, S.-Y.; Park, S.-J., Highly Porous Carbon Aerogels for High-Performance Supercapacitor Electrodes. *Nanomaterials* **2023,** 13, (5), 817.
2. Karaaslan, M. A.; Lin, L.-T.; Ko, F.; Renneckar, S., Carbon Aerogels From Softwood Kraft Lignin for High Performance Supercapacitor Electrodes. *Frontiers in Materials* **2022,** 9, 894061.
3. Luo, M.; Wang, X.; Meng, T.; Yang, P.; Zhu, Z.; Min, H.; Chen, M.; Chen, W.; Zhou, X., Rapid one-step preparation of hierarchical porous carbon from chitosan-based hydrogel for high-rate supercapacitors: The effect of gelling agent concentration. *International Journal of Biological Macromolecules* **2020,** 146, 453-461.
4. Bhartiya, S.; Singh, R.; Singh, A.; Balal, M.; Bhardwaj, P.; Kohli, D. K.; Singh, M. K., Nitrogen-doped carbon aerogel synthesis by solvothermal gelation for supercapacitor application. *Journal of Solid State Electrochemistry* **2022,** 26, (12), 2829-2839.
5. Ping, Y.; Yang, S.; Han, J.; Li, X.; Zhang, H.; Xiong, B.; Fang, P.; He, C., N-self-doped graphitic carbon aerogels derived from metal–organic frameworks as supercapacitor electrode materials with high-performance. *Electrochimica Acta* **2021,** 380, 138237.
6. Lv, Y.; Ding, L.; Wu, X.; Guo, N.; Guo, J.; Hou, S.; Tong, F.; Jia, D.; Zhang, H., Coal-based 3D hierarchical porous carbon aerogels for high performance and super-long life supercapacitors. *Scientific Reports* **2020,** 10, (1), 7022.
